# A Retrospective Study of Clinical and Genetic Features in a Long-Term Cohort of Mexican Children with Alagille Syndrome

**DOI:** 10.3390/ijms26157626

**Published:** 2025-08-06

**Authors:** Rodrigo Vázquez-Frias, Gustavo Varela-Fascinetto, Carlos Patricio Acosta-Rodríguez-Bueno, Alejandra Consuelo, Ariel Carrillo, Magali Reyes-Apodaca, Rodrigo Moreno-Salgado, Jaime López-Valdez, Elizabeth Hernández-Chávez, Beatriz González-Ortiz, José F Cadena-León, Salvador Villalpando-Carrión, Liliana Worona-Dibner, Valentina Martínez-Montoya, Arantza Cerón-Muñiz, Edgar Ramírez-Ramírez, Tania Barragán-Arévalo

**Affiliations:** 1 Subdirección de Investigación, Hospital Infantil de México Federico Gómez, Mexico City 06720, Mexico; rovaf@yahoo.com; 2 Departamento de Trasplantes, Hospital Infantil de México Federico Gómez, Mexico City 06720, Mexico; 3 Departamento de Gastroenterología, Hospital Infantil de México Federico Gómez, Mexico City 06720, Mexico; 4 Hospital Infantil de México Federico Gómez, Mexico City 06720, Mexico; 5 Unidad de Investigación y Diagnóstico en Nefrología y Metabolismo Mineral Óseo, Hospital Infantil de México Federico Gómez, Mexico City 06720, Mexico; 6 Departamento de Genética, Hospital Infantil de México Federico Gómez, Mexico City 06720, Mexico; 7 Centenario Hospital Miguel Hidalgo, Aguascalientes 20000, Mexico; 8 Servicio de Gastroenterología y Nutrición Pediátrica, UMAE, Hospital de Pediatría Centro Médico Nacional de Occidente, Instituto Mexicano del Seguro Social, Guadalajara 44160, Mexico; 9 Hospital Pediatría Centro Médico Nacional Siglo XXI, Instituto Mexicano del Seguro Social, Mexico City 06720, Mexico; 10 Instituto Nacional de Pediatría, Mexico City 04530, Mexico; 11 Biopas a Swixx BioPharma Company, Mexico City 11000, Mexico; 12 Aequitas Medica, Mexico City 03810, Mexico

**Keywords:** Alagille syndrome, case series, *JAG1* gene, *NOTCH2* gene, genetic variant, phenotype

## Abstract

Alagille syndrome (ALGS) is a multisystem disorder characterized by a paucity of intrahepatic bile ducts and cholestasis, often requiring liver transplantation before adulthood. Due to the lack of genotype–phenotype correlation, case series are essential to understand disease presentation and prognosis. Data on Mexican ALGS patients are limited. Therefore, we aimed to characterize a large series of Mexican patients by consolidating cases from major institutions and independent geneticists, with the goal of generating one of the most comprehensive cohorts in Latin America. We retrospectively analyzed clinical records of pediatric ALGS patients, focusing on demographics, clinical features, laboratory and imaging results, biopsy findings, and transplant status. Genetic testing was performed for all cases without prior molecular confirmation. We identified 52 ALGS cases over 13 years; 22 had available clinical records. Of these, only 6 had molecular confirmation at study onset, prompting genetic testing in the remaining 16. We identified six novel *JAG1* variants and several previously unreported phenotypic features. A liver transplantation rate of 13% was observed in the cohort. This study represents the largest molecularly confirmed ALGS cohort in Mexico to date. Novel genetic and clinical findings expand the known spectrum of ALGS and emphasize the need for improved therapies, such as IBAT inhibitors, which may alleviate symptoms and reduce the need for transplantation.

## 1. Introduction

Alagille syndrome (ALGS; MIM118450) is an autosomal dominant multisystem disorder caused predominantly by pathogenic variants in *JAG1* and, less frequently, in *NOTCH2* genes, with a de novo occurrence rate of 60–70% [1,2,3,4]. ALGS is primarily characterized by a paucity of intrahepatic bile ducts and cholestasis, often accompanied by cardiac anomalies, skeletal defects, ocular abnormalities, and distinctive facial features [1,5,6,7]. Renal involvement and vascular malformations are also common [1]. The estimated incidence ranges from 1 in 30,000 to 1 in 70,000 live births [8]. Approximately 2% of cases test negative for variants in these genes [5]. Large deletions involving the *JAG1* gene have also been described in patients with this disease [9]. The syndrome is named after the French pediatric hepatologist Daniel Alagille [10], though its earliest description is attributed to MacMahon in 1948 [10,11].

Management of ALGS is primarily supportive, aimed at controlling cholestasis-related complications and optimizing nutritional status. Pruritus is often the most debilitating symptom [1]. Therefore, reducing the bile load is one of the mainstays of treatment. Ursodeoxycholic acid is commonly used to promote bile excretion [6]. However, in patients affected by refractory pruritus, surgical procedures become necessary, with partial external biliary diversion as the most common procedure [6,12]. Liver transplantation is commonly performed as treatment for cholestasis-related complications, including intractable pruritus [5]. While up to 90% of patients survive childhood, only 40% retain their native liver by adulthood [5,13].

Several medications, such as ursodeoxycholic acid, cholestyramine, rifampicin, and selective serotonin reuptake inhibitors, have been used off-label to manage pruritus, targeting different mechanisms of action [6,14]. However, their efficacy remains limited. More recently, ileal bile acid transporter (IBAT) inhibitors have since been developed to inhibit the enterohepatic recirculation of bile acids, offering a targeted approach that reduces hepatic burden and effectively manages pruritus in ALGS patients [3,15].

ALGS exhibits variable expressivity and incomplete penetrance. This is associated with no clear genotype–phenotype correlations, making it difficult to predict the course of the disease [2,6,16,17]. This has critical implications in clinical practice. On one hand, this complicates diagnosis, as a significant proportion of patients fail to meet classic diagnostic criteria. On the other hand, it limits the ability to provide accurate prognosis [1]. Case series play a crucial role in this regard, providing aggregated insights that inform clinical expectations and management strategies.

In Mexico, data on the clinical characteristics of ALGS are limited to a few small series [18,19]. To address this gap, we conducted a retrospective analysis of an expanded cohort drawn from multiple institutions, including genetic testing in all patients without prior molecular confirmation. This study combines data from a previously published series [18] with newly identified cases from national centers and independent clinical geneticists, totaling 22 children with molecularly confirmed ALGS. While phenotypic features were largely consistent with global cohorts, we observed a notably lower molecular confirmation and liver transplantation rate compared with international reports. This finding underscores possible gaps in therapeutic access and highlights the need to improve management strategies for ALGS in Mexico. Our cohort represents the largest molecularly confirmed series in the country and one of the most comprehensive in Latin America, offering valuable insights into the local disease profile and treatment challenges.

## 2. Results

**Figure 1:**
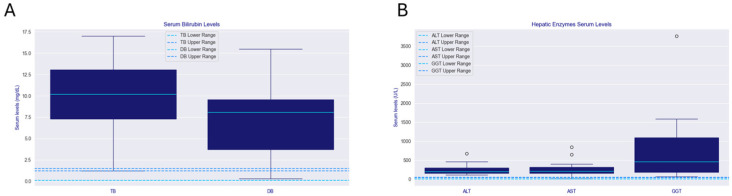
Box plots of liver function tests. (**A**) Total bilirubin and direct bilirubin in ALGS patients. (**B**) Hepatic enzymes in ALGS patients. The dashed lines represent the upper and lower normal ranges of these parameters. ALT = alanine aminotransferase; AST = aspartate aminotransferase; DB = direct bilirubin; GGT = gamma-glutamyl transferase; TB = total bilirubin.

**Figure 2:**
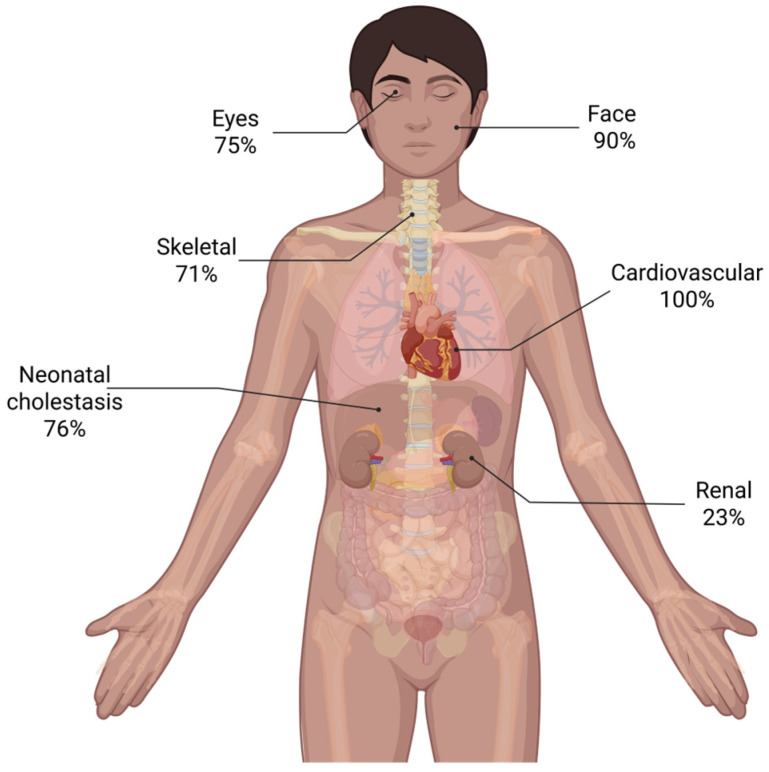
Frequency of phenotypic characteristics of patients with ALGS. Created in BioRender.com (accessed on 11 April 2024).

Full demographic and clinical data are summarized in Table 1. Laboratory findings based on available information on 14 patients are displayed in Figure 1. Table 2 displays the liver biopsy findings of 12 patients. Table 3 displays sequence variants found in *JAG1* and *NOTCH2* genes. Figure 2 summarizes phenotypical findings in patients.

### 2.1. Study Population

A total of 52 patients diagnosed with ALGS were identified through registry screening over a 13-year period. However, complete clinical records were available for only 22 patients, who were subsequently included in the study cohort. Of these, seventeen were male and five female, yielding a male-to-female ratio of approximately 3:1. A selection of pedigrees is displayed in Figure S1. The median age at onset was 8 weeks (range: 1–12 weeks), and the median age at diagnosis was 12 weeks (range: 1 week–4 years). Four patients had a family history of ALGS. At the time of this report, three patients had undergone liver transplantation.

### 2.2. Clinical Manifestations

**Figure 3:**
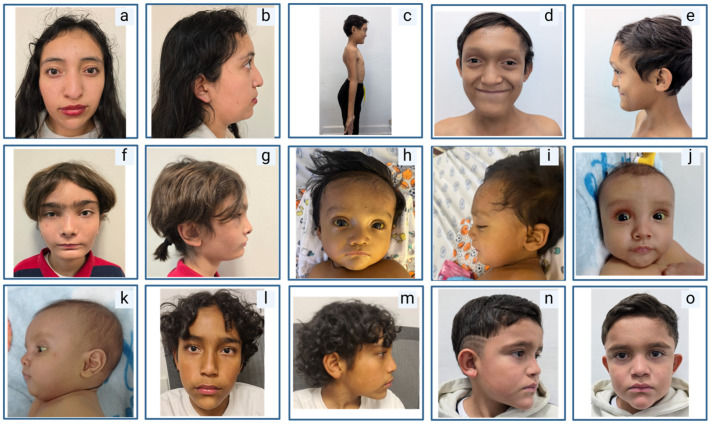
Characteristic facial features of patients with ALGS. (**a**,**b**) Notice the normal phenotype of one of the patients. (**c**–**e**) Patient with a prominent forehead, hypertelorism, and a triangular chin; additionally, this patient had osteopenia. (**f**,**g**) Patient with a broad forehead, enophthalmos, and a pointed chin. (**h**,**i**) Patient with a prominent forehead, hypertelorism, and a triangular chin. (**j**,**k**) Patient with a pointed chin. (**l**,**m**) Patient with a prominent forehead, hypertelorism, and a triangular chin. (**n**,**o**) Patient with a prominent forehead, hypertelorism, and a triangular chin. Created in BioRender.com.

Neonatal cholestasis was observed in 76% of patients; in one case, it was accompanied by intrahepatic portal hypertension. Esophageal varices were noted in a single case (5%). Laboratory findings for liver function tests are summarized in Figure 1. Cardiovascular anomalies were identified in all patients, with pulmonary artery stenosis (47%), pulmonary artery hypoplasia (20%), and patent ductus arteriosus (13%) being the most common; less frequent anomalies (7% each) included septal defects, patent foramen ovale, tetralogy of Fallot, tricuspid insufficiency, pulmonary valve agenesis, and crossed pulmonary arteries. Renal abnormalities were observed in 23% of patients, comprising left renal dysplasia with a right solitary kidney, a double collecting system, and renal tubular acidosis. Skeletal defects occurred in 71% of patients, predominantly butterfly vertebrae (50%), with isolated cases of hemivertebrae and scoliosis. Ophthalmologic anomalies were present in 75% of patients, with posterior embryotoxon as the most frequent finding (52%). Rare anomalies (6% each) included blue sclerae, retinal pigmentary changes with right exotropia, Lisch nodules, and visual immaturity due to delayed conduction; some co-occurred with posterior embryotoxon. Characteristic facial features (deep-set eyes, broad forehead, up-slanting palpebral fissures, straight nose with bulbous tip, and pointed chin) were identified in 90% of patients. A collection of pictures depicting facial characteristics of a selection of patients is displayed in Figure 3. Other rare findings included a Müllerian anomaly type 1 with reduced-size ovaries in one patient, mild motor delay in another, and a combination of cleft lip and palate, hypospadias, cryptorchidism, and conductive hearing loss in a third.

### 2.3. Histopathology

Liver biopsy data were available for 12 patients. Intrahepatic bile duct paucity was observed in seven cases (59%). Evidence of cholestasis was found in six patients (50%), and four patients (33%) exhibited fibrotic changes. Lobular disarray was identified in three patients (25%), and evidence of portal hypertension was noted in two patients (17%). One patient showed no significant changes in the liver biopsy. Detailed biopsy findings are summarized in Table 2.

### 2.4. Genetic Findings

**Figure 4:**
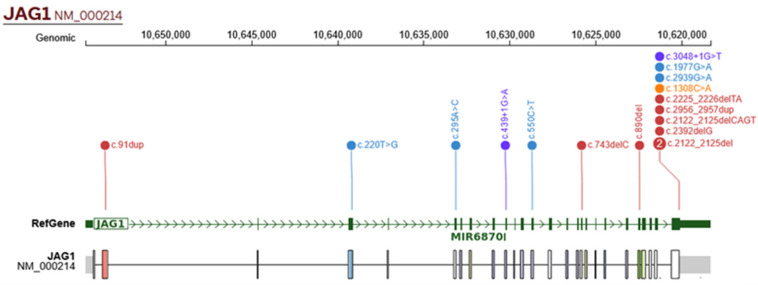
Sequence variants of *JAG1* gene in patients with ALGS. Missense variants are represented in blue; frameshift variants are represented in red; nonsense variants are represented in orange; and splice-site variants are represented in purple. Created with Protein Paint [20].

At the time of inclusion, only 6 out of 22 patients had a genetic test confirming an alteration in either the *JAG1* or *NOTCH2* genes. Consequently, 16 patients underwent genetic testing. Seventeen carried pathogenic sequence variants in the *JAG1* gene, while only one exhibited a pathogenic sequence variant in the *NOTCH2* gene. Additionally, P2 exhibited a deletion of exons 1–26 of the *JAG1* gene, detected by NGS panel sequencing; P6 exhibited a deletion at 20 p12.2, detected by chromosomal microarray; P9 exhibited a deletion at 20 p11.2, detected by G-band karyotyping; and a 454.7 kb deletion in chr20 p12.2 was detected in P22 spanning the *JAG1* gene. No patients without pathogenic variants in these genes were identified. A total of 17 distinct pathogenic *JAG1* gene variants were identified: 5 missense, 2 nonsense, 9 frameshift, and 2 splice-site variants. Six novel pathogenic variants in *JAG1* were identified. Sequence variants in *JAG1* are displayed in Table 3, with a visual representation in Figure 4.

### 2.5. Treatment and Outcomes

At the time of this report, 19 patients were under follow-up and alive. P11 and P21 had passed away by the time of this report due to unreported causes. P14 died two weeks after liver transplantation from procedure-related complications. Among patients awaiting liver transplantation, two of three experienced pruritus despite treatment with cholestyramine.

## 3. Discussion

This study, reporting on 22 children diagnosed with ALGS across multiple medical centers with cooperation from independent physicians, underscores the variability in clinical presentation of this condition. To the best of our knowledge, this is the largest cohort of Mexican patients with a molecularly confirmed diagnosis of ALGS reported to date. Unfortunately, clinical records of 30 additional patients were unavailable due to the lack of electronic medical records and routine physical archive purging practices.

### 3.1. Hepatic Manifestations

Our cohort exhibited a slightly lower rate of neonatal cholestasis (76%) compared to most reports in the literature, which typically describe an incidence exceeding 94% [7,19,21]. However, our findings align more closely with the GALA study, which is the largest cohort of ALGS patients to date (82% of cases with neonatal cholestasis) [5]. Additionally, bile duct paucity was documented in 59% of the cases in our study, which is a significantly lower proportion compared with the 70–90% reported in other studies [7,21]. The reason for this discrepancy remains unclear but may reflect sampling bias given the limited number of biopsies performed. Notably, no hepatic lesions such as regenerative nodules or hepatocellular carcinoma were identified in our cohort, which aligns with reports of them being rare findings in patients with ALGS [3]. Interestingly, one patient exhibited no significant histopathological changes in liver biopsy.

### 3.2. Extrahepatic Manifestations

Cardiovascular anomalies were highly prevalent in our study, affecting all assessed patients. Literature reports vary, with Yan et al. describing cardiovascular abnormalities in 75% of patients, Ruiz-Castillo et al. in 100%, and the GALA study in 91% [5,7,19]. Saleh’s classical series noted over 90% incidence of structural cardiac anomalies, with tetralogy of Fallot being among the least frequent at approximately 7% [1].

Skeletal abnormalities were also high in our study (71%), with 50% presenting butterfly vertebrae. Skeletal abnormalities have a variable prevalence across the literature. Series such as that of Liu et al. have a lower rate of skeletal abnormalities, whereas others such as that of Yan et al. have similar rates of these abnormalities [7,21]. The Notch signaling pathway’s role in neural tube development is well recognized [17]. However, findings associated with these anomalies, such as spina bifida occulta were not found in our cohort. Nonetheless, it is important to remember that neural tube defects may represent the first manifestations of ALGS [17].

Posterior embryotoxon is also a frequent finding among patients with ALGS, with a prevalence of 52% in our series. Yan et al. found a similar prevalence with 58% of patients in their series, as well as Cho et al. in 53% of their series [7,22]. However, posterior embryotoxon is not a pathognomonic feature; for example, Chen et al. did not identify it in any patients, while Semenova et al. only found 1 patient among 12 with this anomaly [16,23]. However, it is not pathognomonic, as it has a general population prevalence of up to 15% and does not impair vision [1].

### 3.3. Notable Phenotypes

There are several notable phenotypical findings in our cohort. For instance, P7 exhibited reduced ovarian size and a Müllerian duct anomaly. To the best of our knowledge, such malformations have not been previously reported in patients with ALGS, although the c.1308C>A variant (rs764485729) has been previously described in the literature [22]. Our reason to believe such anomalies are related to the genotype is that the Notch signaling components have been found expressed in the oviducts and uteri of mice [24]. Moreover, animal experiments involving deletion of *JAG1* have demonstrated the development of ovaries with multi-oocytic follicles, reduced granulosa cell proliferation, and increased apoptosis, ultimately leading to subfertility [24]. Additionally, the Notch pathway has also been implicated in ovarian angiogenesis, and its disruption may impair follicular development [25]. While these findings do not constitute direct evidence of a causal relationship between ALGS and the reproductive anomalies observed in this patient, a possible association cannot be ruled out. Notably, reproductive tract malformations are not typically reported in ALGS case series. Future studies could benefit from systematic evaluation of reproductive anatomy in ALGS patients to determine whether such anomalies are underrecognized features of the syndrome.

Two distinct ophthalmological findings are also noticeable. In P13, blue sclerae were noted during examination. This feature is most commonly associated with Osteogenesis Imperfecta [26]. While the sclera does not truly change color, a collagen defect leads to thinning and increased translucency, resulting in a bluish appearance [26]. P22 exhibited a delay in peripheral visual conduction. Hingorani et al. reviewed the ophthalmological manifestations in ALGS patients, but blue sclerae or conduction delays were not identified [27]. To the best of our knowledge, these features have not been reported in more recent cohorts. Although the etiology of these findings remains unclear, and we cannot establish a direct link to ALGS pathogenesis, further investigation may be warranted. Determining the underlying mechanisms is beyond the scope of the present study.

Finally, only one of our patients had a pathogenic variant in *NOTCH2*. Although a genotype–phenotype relation has not been demonstrated among *JAG1* patients, a clear distinction is evident between patients with pathogenic variants in *JAG1* and *NOTCH2* [3], in addition to the overrepresentation of patients affected with *JAG1* variants compared to *NOTCH2* variants [3]. The only patient with a *NOTCH2* variant (P21) displayed a complex genotype that includes cleft lip and palate, hypospadias, cryptorchidism, and conductive hearing loss. Hypospadias and cryptorchidism are common findings in patients affected by *NOTCH2* variants, compared to those with *JAG1* variants [28,29]. Interestingly, the Jagged–Notch signaling pathway is required for the adequate organogenesis of inner-ear bones, leading to conductive hearing loss in some patients [30]. This may explain the conductive hearing loss in this patient.

### 3.4. Genetic Findings

All patients in this cohort were genetically evaluated. Among the 17 patients with pathogenic sequence variants in *JAG1*, 23% were missense, 11% nonsense, 52% frameshift, and 11% splice-site variants. Comparing these results with those of other studies, such as Semenova et al., who found 6% missense, 12% nonsense, 59% frameshift, and 23% splice-site variants, or Yan, who found, 15% missense, 23% nonsense, 46% frameshift, and 8% splice-site variants, reinforces the loss of function as a key pathogenic mechanism for the development of ALGS [7,16].

Notably, P17 and P18 shared the same variant, as they were family-related. However, P15 had a family history of a cousin with an ALGS-compatible phenotype. Unfortunately, it was not possible to include this patient in this series. Overall, 21 distinct sequence variants were detected, including 6 novel pathogenic variants in *JAG1*. As is common in other series, most cases are caused by variants in *JAG1*; our series only found a single case caused by a *NOTCH2* variant [2,6,16]. In our series, four patients had large CNVs encompassing the *JAG1* gene, which were detected either by sequencing or molecular karyotyping. Most notably, one of these patients had a deletion which was large enough to be detectable by G-band karyotyping. Although less frequent, large variants have been previously reported, such as in Lalani et al., albeit with a more complex and variable phenotype [31].

### 3.5. Perspectives

As the phenotypic criteria of ALGS have been expanded, the diagnosis can now be established based on the presence of three out of seven clinical features (hepatic, cardiovascular, renal, skeletal, vascular, ophthalmic manifestations, or characteristic facial features) even without a molecular diagnosis [1,32]. Moreover, the presence of two of these criteria, along with a first-degree relative with confirmed ALGS, may be sufficient to establish the diagnosis [1]. Although genetic testing is not required in these cases, our group strongly recommends targeted testing of *JAG1* and *NOTCH2*.

Our results indicate that Mexican patients with ALGS have no significant genetic or phenotypic differences from other populations. However, the critical difference lies in the accessibility of timely diagnosis and multidisciplinary treatment for ALGS in Mexico. Rare genetic diseases are often characterized by a prolonged “diagnostic odyssey,” typically lasting 5 to 10 years, during which patients may experience health deterioration and missed treatment opportunities [33]. In Mexico, data from the Mexican Rare Disease Patient Registry reveal that the average time for diagnosis in patients with rare diseases is 8 years, with the longest time to diagnosis being 34 years [34]. While NGS has shortened this timeline by up to a half, progress in Mexico remains limited due to systemic healthcare challenges and the absence of standardized national guidelines [33]. This is particularly relevant for multisystemic conditions like ALGS, where early recognition across specialties is essential.

In Mexico, only 17% of patients with rare disorders have received a definitive molecular diagnosis [33]. Early and accurate diagnosis is crucial not only for patients and their families but also for optimizing healthcare resources [33]. Broad implementation of NGS across Mexico could significantly improve diagnostic timelines, not only for ALGS, but for rare genetic diseases more broadly, facilitating access to appropriate care, genetic counseling, clinical trials, and support networks [33]. Although the median age of diagnosis in our cohort was 12 weeks, some patients experienced delays of up to four years. Furthermore, prior to this study, only 6 of 22 patients had received a confirmatory genetic test, underscoring the low accessibility to proper genetic testing in Mexico. These findings underscore the importance of incorporating genetic testing early in the diagnostic process for patients presenting with features of ALGS to facilitate accurate and timely diagnosis and improve clinical outcomes.

Although there is limited information on the therapy of patients in our cohort, the primary therapeutic agents for pruritus were ursodeoxycholic acid and cholestyramine, due to their anticholestatic and antipruritic properties. However, the efficacy of these agents in controlling pruritus remains limited [15]. A recent meta-analysis involving >2000 patients across 32 studies concluded that ursodeoxycholic acid reduced serum bile acid levels by approximately 25.68 μmol/L [35]. To the best of our knowledge, the quantitative reduction in serum bile acids in patients with ALGS treated with cholestyramine has not been studied. In contrast, IBAT inhibitors hold promise for more effective symptom management. Results from the ICONIC trial, which evaluated the efficacy of an IBAT inhibitor in children with ALGS, demonstrated a reduction in serum bile acid levels of 88 μmol/L after 18 weeks of treatment, with reduction reaching up to 181 μmol/L at 204 weeks of treatment, leading to a significant reduction in pruritus [15]. Moreover, by inhibiting enterohepatic recirculation and promoting fecal bile acid excretion, IBAT inhibitors have also been shown to effectively reduce xanthomas, improve growth in children with short stature, and enhance overall quality of life [15].

As IBAT inhibitors are currently unavailable in Mexico, surgical interventions are often necessary for symptom management in ALGS patients. These interventions aim to interrupt the enterohepatic circulation of bile acids or, in refractory cases, involve liver transplantation [15]. Although ALGS is a rare disease, it remains an important indication for pediatric liver transplantation, accounting for an estimated 3–5% of transplants in children [36,37,38]. Long-term studies have estimated that up to 40% of patients can preserve their native liver into adulthood [5]. However, among those who do not undergo liver transplantation, mortality approaches 10% by the age of 18 [5]. Moreover, some series report even lower transplantation rates, closer to the rate of 25% [3]. In our cohort, only 3 out of 22 patients (13%) had received a liver transplant, while 10 of them were on a waiting list for transplantation. This contrasts with global data indicating that approximately 25% of patients with ALGS undergo liver transplantation [5]. In Mexico, during the study period, a total of 2383 liver transplants were performed, including 276 in 2024. Over the same timeframe, 4138 individuals were registered on liver transplant waiting lists, with 239 added in 2024 alone [39]. Moreover, only 10–15% of these transplants were performed in children [40]. Among the 13 patients in our cohort who required a liver transplant, only 23% received one, which is considerably lower than the 57% transplant rate among all patients on the national waiting list. These findings highlight a significant gap in care, suggesting that patients with ALGS may be underrepresented in liver transplant programs or face barriers to access, while also reflecting the need for novel treatments.

IBAT inhibitors represent a promising therapeutic approach for ALGS patients by inhibiting enterohepatic recirculation of biliary acids and enhancing their fecal excretion [15]. These mechanisms result in clinically meaningful improvement in pruritus in over 80% of patients but also contribute to an increased transplant-free survival rate of more than five years [15]. A recent comparison of the GALA study cohort with patients treated with IBAT inhibitors demonstrated a 67% reduction in transplant-free survival [41]. Given that pruritus is the leading indication for liver transplant in ALGS, effective symptom control, alongside potential benefits such as reduced hepatic toxicity, may substantially decrease the need for surgical interventions, including transplantation, in this patient population [41].

### 3.6. Strengths and Limitations

Our study is strengthened by the collaboration of multiple centers and independent clinical geneticists, as well as the extended timeframe over which data were collected. To the best of our knowledge, this series represents the largest cohort of ALGS patients with molecular confirmation reported to date in the Mexican population. In addition, we identified several phenotypical features that are uncommon in patients with ALGS. For example, alterations of the female reproductive tract are not considered a diagnostic criterion for ALGS and, thus, are not routinely assessed. Nonetheless, there is some theoretical evidence suggesting a potential association between *JAG1* variants and this finding. Regarding ophthalmological findings, although posterior embryotoxon is a well-established feature of ALGS, two less common findings were observed in our cohort. A patient presented with blue sclerae, which indicates a thinning of the tissue due to a collagen defect. However, its relationship with *JAG1* alterations remains uncertain. Another patient exhibited a delay in peripheral visual conduction; notably, this patient exhibited a variant of the *NOTCH2* gene. As such, this peripheral visual conduction delay may be attributable to one or more of the affected genes. Additionally, we identified six novel pathogenic or likely pathogenic variants in *JAG1*, further expanding the mutational spectrum associated with ALGS.

Our study is not without limitations. The retrospective design limits the ability to capture detailed information on relevant clinical features, resulting in a partial phenotypic characterization. Also, the absence of centralized and standardized data collection introduces variability in imaging, laboratory, and pathological evaluations. Although this study reflects a collaborative effort among multiple national centers and independent practitioners in México, the lack of interinstitutional communication and the absence of a national registry of ALGS patients constrain both the sample size and the depth of clinical characterization. To address these challenges, future research should adopt a prospective design, implementing standardized diagnostic protocols, and be supported by a coordinated, nation-wide strategy. Such efforts would provide a better understanding of the clinical and genetic landscape of ALGS in México. Moreover, it would provide a critical foundation for assessing the efficacy of emerging therapies, particularly IBAT inhibitors, which have shown promise in reducing pruritus, reducing the need for transplantation and improving quality of life in ALGS patients. As these treatments gain regulatory approval and enter clinical use, robust national data will be essential to evaluate their long-term benefits and inform equitable access and clinical decision-making.

## 4. Materials and Methods

### 4.1. Study Design, Population, and Data Collection

This study was designed as a retrospective analysis of clinical records of pediatric patients diagnosed with ALGS across multiple institutions. The study population included children diagnosed with ALGS based on clinical criteria, liver biopsy, or both, who had genetic testing of *JAG1* and *NOTCH2* genes, and who were admitted to any of the collaborating institutions between 2012 and 2025. Clinical data were collected when available, including demographic data (age, sex, geographic origin), affected family members, serum bilirubin and hepatic enzymes levels at onset, liver biopsy findings, and skeletal, ophthalmological, renal and vascular assessments, as well as the presence of certain facial features and liver transplantation status.

### 4.2. Genetic Analysis

At the start of data collection (November 2024), patients with a clinical diagnosis of ALGS who had not undergone prior genetic testing were analyzed using a next-generation sequencing (NGS) panel provided by the study sponsor through an external laboratory (Mendelics, São Paulo, Brasil). This panel comprised 17 genes associated with ALGS and progressive familial intrahepatic cholestasis, with *JAG1* and *NOTCH2* among the targets, and was designed using a customized capture kit (Twist Bioscience, San Francisco, CA, USA), covering >99% of the targeted regions at >10× depth. Sequencing was performed on an Illumina NovaSeq 6000 platform (Illumina Inc., San Diego, CA, USA), and reads were aligned to the human reference genome (GRCh38). Variants in ALGS-associated genes were interpreted according to the American College of Medical Genetics and Genomics (ACMG) classification criteria [42].

For patients who had previously undergone genetic testing, methodologies varied across centers and included NGS panel sequencing, clinical exome sequencing, molecular karyotyping (array-based comparative genomic hybridization or SNP arrays), and conventional G-band karyotyping. The choice of technique depended on each institution’s technical resources and the preferences of attending geneticists. Variant confirmation was performed according to the internal protocols of the respective commercial laboratories. These confirmation workflows included methods such as Sanger sequencing, multiplex ligation-dependent probe amplification (MLPA), MLPA-seq, or long-read sequencing technologies. Only P9′s karyotyping was performed in house at Hospital Infantil de México; all other analyses were carried out in external laboratories.

### 4.3. Liver Histopathology

Liver biopsy was performed on patients according to the attending physician’s direction and institutional protocols. Histological examination included hematoxylin and eosin (H&E) staining. Bile duct paucity was defined as the absence of interlobular bile ducts in ≥50% of portal tracts in biopsy samples containing ≥10 portal areas.

### 4.4. Other Assessments

Cardiac abnormalities were evaluated through echocardiography for identifying congenital heart defects such as pulmonary stenosis, atrial septal defects, and ventricular septal defects. Vertebral anomalies were assessed via spinal X-rays, and ophthalmic abnormalities were identified using slit-lamp examination. Abdominal ultrasound and MRI were used to evaluate hepatobiliary involvement, including signs of cirrhosis, portal hypertension, and splenomegaly. Patients who received a liver transplant or were on a waiting list for transplantation were noted.

### 4.5. Statistical Analysis

Data from clinical records was initially entered into a Microsoft Excel spreadsheet (Redmond, WA, USA: Microsoft, v2407) and exported to a CSV file. Descriptive statistics were used to summarize demographic and clinical data. Continuous variables were presented as medians and interquartile ranges (IQRs), while categorical variables were expressed as frequencies and percentages. Statistical analyses were conducted using Python (Python Language Reference, version 3.12. Wilmington, DE, USA: Python Software Foundation, 2023), with the SciPy v. 1.15.3, NumPy v. 2.2.5, and Pandas v. 2.2.3 libraries employed for computations and Matplot v. 0.1.9, Seaborn 0.13.2, and StatAnnotations v. 0.7.2 libraries for data visualization.

### 4.6. Ethical Considerations

This study was approved by the ethics committee of Hospital Infantil de Mexico Federico Gomez (protocol number and bioethics approval: HIM/2024/065, approved on 10 December 2024), with the rest of the institutions and independent clinicians adhering to this protocol and ethics guidelines. Clinicians involved in this study were required to obtain informed consent from patients or their guardians before submitting their medical records and images for this study. Informed consent was obtained from patients’ parents or legal guardians before genetic testing, liver biopsy, and any invasive procedures. All patients or their guardians signed consent for publication of anonymized clinical data and photographs. All procedures followed the principles of the Declaration of Helsinki.

## 5. Conclusions

ALGS is a rare multisystemic disorder with no clear genotype–phenotype correlation. Our cohort represents the largest series of Mexican patients with molecular confirmation reported to date. At the genetic level, major findings include six novel variants in the *JAG1* gene, a deletion spanning several genes with a complex phenotype and a large deletion detectable through G-band karyotyping. Phenotypical findings include previously unreported features such as female reproductive tract anomalies, visual disturbances (including blue sclerae and peripheral visual conduction delay), hypospadias, and cryptorchidism. Additionally, we observed features such as conductive hearing loss and cleft lip and palate, which, though previously described, further expand the recognized phenotypic spectrum of ALGS. Given the lack of a genotype–phenotype correlation, population-specific case series provide valuable insights for clinicians and researchers. Importantly, our findings highlight the urgent need for effective therapeutic interventions targeting hepatic manifestations. Nearly half of our cohort remains on the waiting list for liver transplantation, underscoring the need for novel treatments and the potential role of novel IBAT inhibitors in alleviating pruritus, improving liver function in affected individuals, and reducing the need for hepatic transplantation.

## Figures and Tables

**Table 1:** Demographic and clinical characteristics of 22 patients with ALGS. Clinical data and demographic data were recorded when available from clinical records.

Patient	Sex	Age at Onset	Age at Diagnosis	Age at Last Follow-Up	Family History	Neonatal Cholestasis (*n* = 17)	Cardiovascular Findings (*n* = 15)	Renal Findings (*n* = 13)	Vascular Findings (*n* = 11)	Skeletal Finding (*n* = 14)	Ophthalmic Findings (*n* = 17)	Facial Features (*n* = 10)	Pruritus (*n* = 5)	Age at Transplant (If Performed) (*n* = 15)
P1	Male	-	1 mo	11 y	No	Yes	PAS	No	No	Butterfly vertebrae	Retinal pigmentary changes, right exotropia	BFH, HypT, PC	-	6 y
P2	Female	3 mo	3 y	19 y	No	Yes	PAS	Double right collecting system	No	Scoliosis	No	-	No	9 y
P3	Male	2 mo	2 mo	9 y	No	Yes	Crossed pulmonary arteries	Left renal atrophy, right solitary kidney	No	Hemivertebra	Posterior embryotoxon	-	-	Unspecified
P4	Male	-	3 mo	2 y	No	Yes, with intrahepatic portal hypertension	VSD	-	No	No	No	-	-	Waiting list
P5	Male	-	3 mo	10 y	Mother with suspected but unconfirmed ALGS	Yes	PAS	No	No	Scoliosis	Posterior embryotoxon	BFH, DSE, PC	No	Waiting list
P6	Male	15 d	NA	2 y	No	Yes	-	-	-	-	-	-	-	Waiting list
P7	Female	-	-	17 y	No	Yes	PA, tricuspid insufficiency	No	-	Butterfly vertebrae	Posterior embryotoxon	Tc, PC	-	Waiting list
P8	Female	1 mo	-	6 y	No	Yes	PAS	No	EV	Butterfly vertebrae	Posterior embryotoxon	BFH, DSE, PC	-	Waiting list
P9	Male	-	-	-	-	-	-	-	-	-	-	-	-	-
P10	Male	-	-	-	-	-	-	-	-	-	-	-	-	-
P11	Male	-	-	Deceased	-	-	-	-	-	-	-	-	-	-
P12	Male	-	-	-	-	-	-	-	-	-	-	-	-	-
P13	Female	-	-	9 y	No	No	ASD	No	-	Butterfly vertebrae	Posterior embryotoxon, blue sclerae, deep-set eyes	-	-	-
P14	Male	1 w	1 w	-	No	Yes	PAS	No	-	Butterfly vertebrae	No	No	-	0 y, deceased 2 weeks after transplant
P15	Male	2 mo	2 mo	6 y	Yes: one parent and a parent’s cousin	Yes	SVPS, PAS, PFO, PDA	VUR, post-operative left UPJ stenosis, RTA	No	Butterfly vertebrae	No	BFH, DSE, PE, BNT, PC	-	Waiting list
P16	Male	2 mo	2 mo	1 y	No	Yes	VSD	No	No	No	No	Dol, BFH, SE, UPF, EF, DSE, BNT	-	Waiting list
P17	Male	15 d	3 y	-	Yes, one of the parents	Yes	PDA	No	No	No	Posterior embryotoxon	SE, UPF, HypT, BNT, TUL, PC, AI, ICHU	-	Waiting list
P18	Female	-	-	1 y	Yes: two paternal cousins deceased at age 2 with unspecified hepatopathy	No	TOF with pulmonary valve agenesis	No	No	Butterfly vertebrae	Posterior embryotoxon	DSE, PC	Yes, on treatment with cholestyramine	Waiting list
P19	Male	-	-	-	-	-	-	-	-	-	Lisch Nodules	-	-	No
P20	Male		4 y	-	No	No	PAS	-	-	-	Posterior embryotoxon	-	Yes, on treatment with ursodeoxycholic acid	-
P21	Male	-	-	Deceased	No	No	-	No	No	No	Posterior embryotoxon	-	-	-
P22	Male	2 mo	5 mo	5 mo	No	Yes	PAS	-	-	-	Visual immaturity due to delayed conduction	BFH, HypT, PC	No	Waiting list

Abbreviations: AI: auricles with adequate implantation; ALGS: Alagille syndrome; ASD: atrial septal defect; BFH: broad forehead; BNT: bulbous nasal tip; D: days; Dol: dolichocephaly; DSE: deep-set eyes; EF: epicanthus; EV: esophageal varices; HypT: hypertelorism; ICHU: inferior crus of helix underdevelopment; Mo: months; No: none reported; P: patient; PAS: pulmonary artery stenosis; PDA: patent ductus arteriosus; PE: prominent ears; PC: pointed chin; PFO: patent foramen ovale; RTA: renal tubular acidosis; SE: sparse eyebrows; SVPS: supravalvular pulmonary stenosis; Tc: telecanthus; TUL: thin upper lip vermilion; TOF: tetralogy of Fallot; UPF: up-slanting palpebral fissures; UPJ: ureteropelvic junction; VSD: ventricular septal defect; VUR: vesicoureteral reflux; Y: years. When data were unavailable, a “-“ was used.

**Table 2:** Liver biopsy findings in 10 patients. Findings are from the evaluating pathologists. The most common finding was bile duct paucity or hypoplasia, with a combination of cholestasis, fibrosis, lobular disarray, and occasional additional findings. Only one patient was reported with no significant changes.

Patient	Liver Biopsy
P1	Reduced portal spaces and bile ducts; no cholangial proliferation
P2	Decreased intrahepatic bile ducts, bile retention in hepatocytes, dilated sinusoids
P3	Intrahepatic bile duct hypoplasia
P4	Partial biliary flow obstruction, stage 3 fibrosis
P7	Decreased interlobular bile ducts
P13	Minimal changes suggestive of portal hypertension secondary to efferent flow obstruction
P14	No significant changes
P16	Giant cell hepatitis, lobular disarray, cholestasis, mild portal fibrosis, no ductular proliferation, no microorganisms detected
P17	Expanded portal spaces with lymphoplasmacytic infiltrate, fibrosis with septal formation, biliary pigment in hepatocytes
P18	Bile duct paucity, giant cell hepatitis
P21	Fibrosis
P22	Bile duct hypoplasia, cholangial proliferation, intracanalicular and intracytoplasmic cholestasis, scattered apoptotic cells

**Table 3:** Genetic sequence variants in *JAG1* and *NOTCH2* genes of 16 patients with ALGS. Large deletions were omitted from this table. Six novel variants were discovered in the *JAG1* gene. Pathogenicity was assessed according to ACGM Standards and Guidelines. Two variants were previously reported in ClinVar, but no article citing these specific variants was found.

Patient	Gene	NM ID	cDNA	Amino Acid Change	rsID	Mutation Type	Classification	Previous Reports in the Literature (PMID)
P1	*JAG1*	NM_000214.3 (*JAG1*)	c.550C>T	p.Arg184Cys	rs121918350	Missense	Likely Pathogenic (PM1, PM2, PM5, PP2, PP3, PP4, PP5)	24748328
P3	*JAG1*	NM_000214.3 (*JAG1*)	c.295A>C	p.Thr99Pro	-	Missense	Likely Pathogenic (PM1, PM2, PM5, PP2, PP3, PP4)	Novel
P4	*JAG1*	NM_000214.3 (*JAG1*)	c.743delC	p.Pro248Glnfs*164	-	Frameshift	Pathogenic (PVS1, PS3, PM2, PP4)	Novel
P5	*JAG1*	NM_000214.3 (*JAG1*)	c.2392delG	p.Val798Trpfs*22	-	Frameshift	Pathogenic (PVS1, PS3, PM2, PP4)	Novel
P7	*JAG1*	NM_000214.3 (*JAG1*)	c.1308C>A	p.Cys436*	rs764485729	Nonsense	Pathogenic (PVS1, PM2, PP2, PP4)	25676721
P8	*JAG1*	NM_000214.3 (*JAG1*)	c.220T>G	p.Tyr74Asp	-	Missense	Likely pathogenic (PM1, PM2, PM5, PP3, PP4)	-
P10	*JAG1*	NM_000214.3 (*JAG1*)	c.2122_2125delCAGT	p.Gln708Valfs*34	rs727504412	Frameshift	Pathogenic (PVS1, PS3, PM2, PP3, PP4, PP5)	25676721
P11	*JAG1*	NM_000214.3 (*JAG1*)	c.439+1G>A	-	rs863223648	Splice donor variant	Pathogenic (PVS1, PM1, PM2, PP3, PP4, PP5)	24748328
P12	*JAG1*	NM_000214.3 (*JAG1*)	c.2939G>A	p.Cys980Tyr	-	Missense	Likely pathogenic (PM1, PM2, PM5, PP2, PP3, PP4)	Novel
P13	*JAG1*	NM_000214.3 (*JAG1*)	c.890del	p.Gly270AspfsTer145	-	Frameshift	Pathogenic (PVS1, PS3, PM1, PM2, PP4)	Novel
P14	*JAG1*	NM_000214.3 (*JAG1*)	c.91dup	p.Ala31Glyfs*42	-	Frameshift	Pathogenic (PVS1, PS3, PM2, PP4).	Novel
P15	*JAG1*	NM_000214.3 (*JAG1*)	c.2956_2957dup	p.Leu986fs	rs2122595849	Frameshift	Pathogenic (PVS1, PS3 PM2, PP4, PP5).	-
P16	*JAG1*	NM_000214.3 (*JAG1*)	c.2122_2125del	p.Gln708Valfs*34	rs727504412	Frameshift	Pathogenic (PVS1, PS3, PM2, PP4, PP5)	15712272
P17	*JAG1*	NM_000214.3 (*JAG1*)	c.2122_2125del	p.Gln708Valfs*34	rs727504412	Frameshift	Pathogenic (PVS1, PS3, PM2, PP4, PP5)	22488849
P18	*JAG1*	NM_000214.3 (*JAG1*)	c.2225_2226delTA	p.Ile742SerfsTer5	rs1555828209	Frameshift	Pathogenic (PVS1, PS3, PM2, PP4, PP5).	21752016
P19	*JAG1*	NM_000214.3 (*JAG1*)	c.1977G>A	p.Trp659*	rs1600182107	Nonsense	Pathogenic (PVS1, PS3, PM1, PM2, PP4, PP5)	12497640
P20	*JAG1*	NM_000214.3 (*JAG1*)	c.3048+1G>T	-	rs876661121	Splice donor variant	Pathogenic (PVS1, PM2, PP3, PP4, PP5)	31343788
P21	*NOTCH2*	NM_024408.4 (*NOTCH2*)	c.3878G>A	p.Arg1293His	rs201968231	Missense	Variant of Uncertain Significance (PM1, PM2, PP4)	-

## Data Availability

The data are not publicly available due to patient privacy concerns. However, anonymized data that support the findings of this study are available upon reasonable request from the corresponding author.

## Supplementary Materials

The following supporting information can be downloaded at: https://www.mdpi.com/article/10.3390/ijms26157626/s1.

## Abbreviations

The following abbreviations are used in this manuscript:

ACMG	American College of Medical Genetics and Genomics
AI	Auricles with adequate implantation
ALGS	Alagille syndrome
ALT	Alanine aminotransferase
ASD	Atrial septal defect
AST	Aspartate aminotransferase
BFH	Broad forehead
BNT	Bulbous nasal tip
CNV	Copy number variant
DB	Direct bilirubin
Dol	Dolichocephaly
DSEs	Deep-set eyes
EF	Epicanthus
EVs	Esophageal varices
GGT	Gamma-glutamyl transferase
H&E	Hematoxylin and eosin
HypT	Hypertelorism
ICHU	Inferior crus of helix underdevelopment
IBAT	Ileal bile acid transporter
MRI	Magnetic resonance imaging
NGS	Next-generation sequencing
PAS	Pulmonary artery stenosis
PDA	Patent ductus arteriosus
PE	Prominent ears
PC	Pointed chin
PFO	Patent foramen ovale
PM	Pathogenic moderate
PP	Pathogenic supporting
PS	Pathogenic strong (ACMG classification evidence code)
PVS	Pathogenic very strong (ACMG classification evidence code)
RTA	Renal tubular acidosis
SVPS	Supravalvular pulmonary stenosis
TB	Total bilirubin
Tc	Telecanthus
TUL	Thin upper lip vermilion
TOF	Tetralogy of Fallot
UPF	Up-slanting palpebral fissures
UPJ	Ureteropelvic junction
VSD	Ventricular septal defect
VUR	Vesicoureteral reflux
