# Supplementary material for: A Retrospective Study of Clinical and Genetic Features in a Long-Term Cohort of Mexican Children with Alagille Syndrome
Source: Int J Mol Sci. 2025 Aug 6;26(15):7626. doi: 10.3390/ijms26157626 (PMC12347795; doi:10.3390/ijms26157626)
Supplement: Supplementary file 1 [file ijms-26-07626-s001.zip › ijms-3716672-supplementary.pdf]

Supplementary file for: Rodrigo Vázquez-Frías, Gustavo Varela-Fascinetto, Carlos Patricio Acosta-Rodríguez-Bueno, Alejandra Consuelo, Ariel Carrillo, Magali Reyes-Apodaca, Rodrigo Moreno-Salgado, Jaime López-Valdez, Elizabeth Hernández-Chávez, Beatriz González-Ortiz, José F Cadena-León, Salvador Villalpando-Carrión, Liliana Worona-Dibner, Valentina Martínez-Montoya, Arantza Cerón-Muñiz, Edgar Ramírez-Ramírez and Tania Barragán-Arévalo. A Retrospective Study of Clinical and Genetic Features in a Long-Term Cohort of Mexican Children with Alagille Syndrome. Int. J. Mol. Sci. 2025.

A

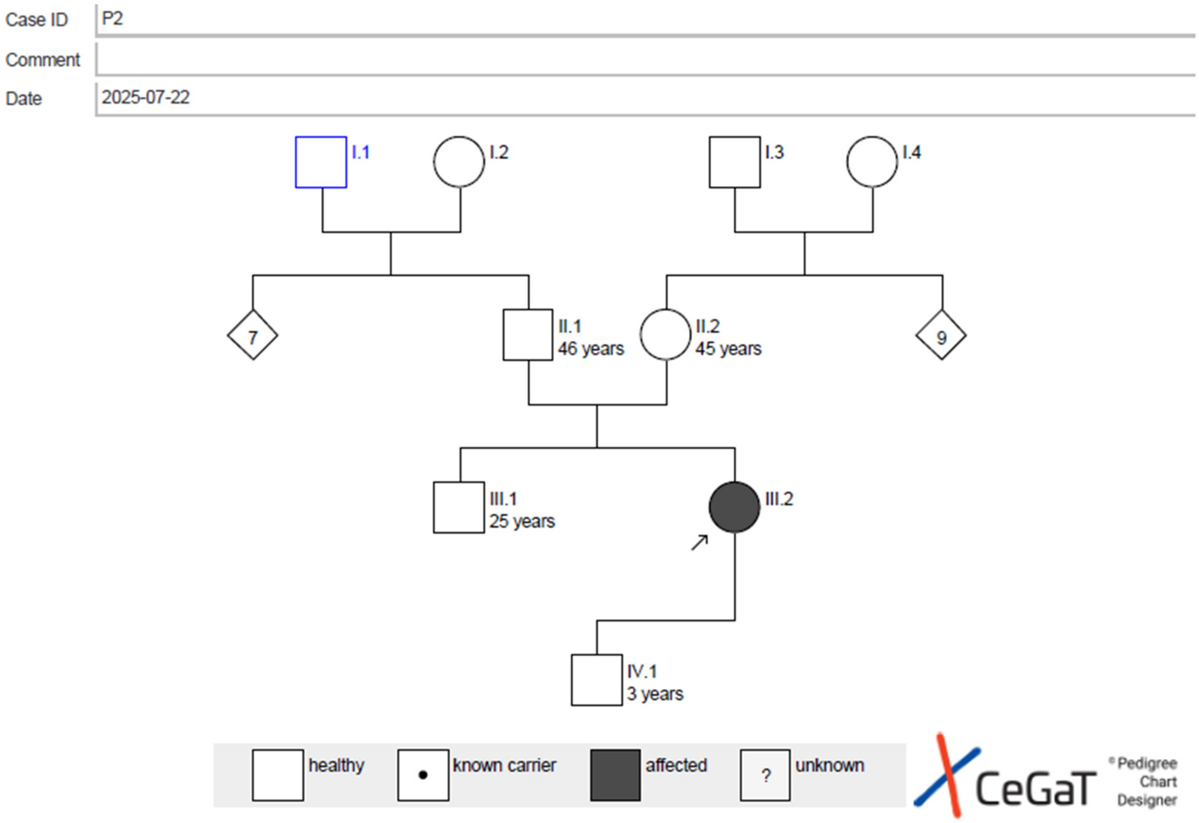

B

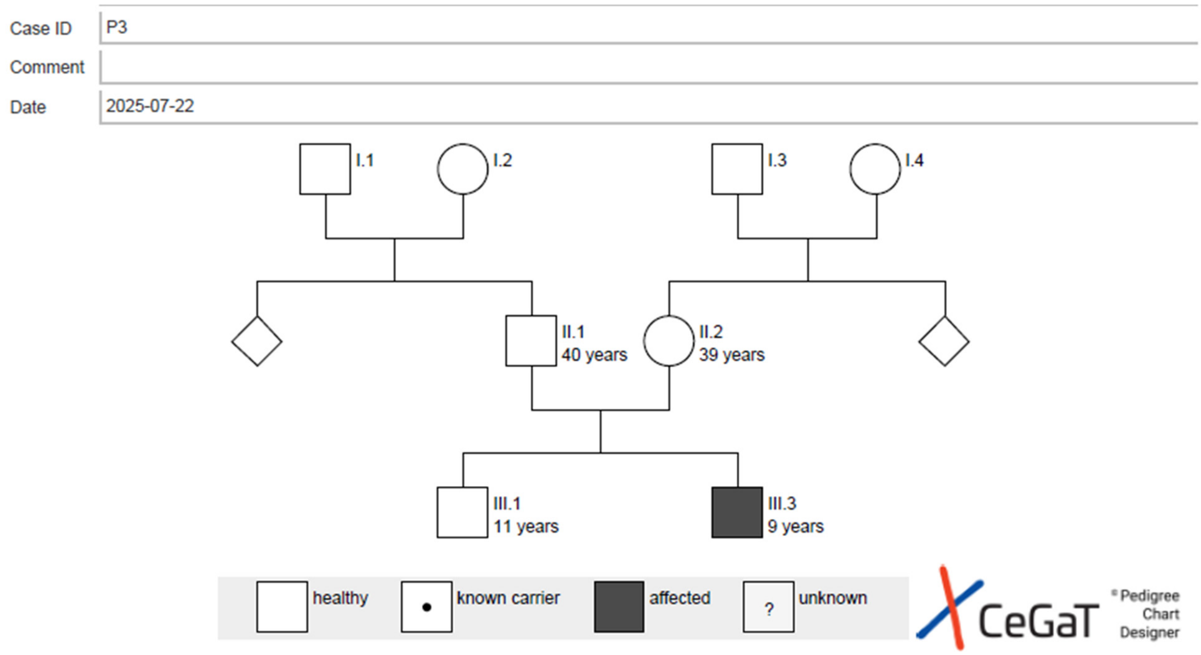

Case ID
P3
Comment
Date
2025-07-22
I.1
I.2
I.3
I.4
II.1
40 years
II.2
39 years
III.1
11 years
III.3
9 years
healthy
known carrier
affected
?
unknown
CeGaT
® Pedigree
Chart
Designer

C

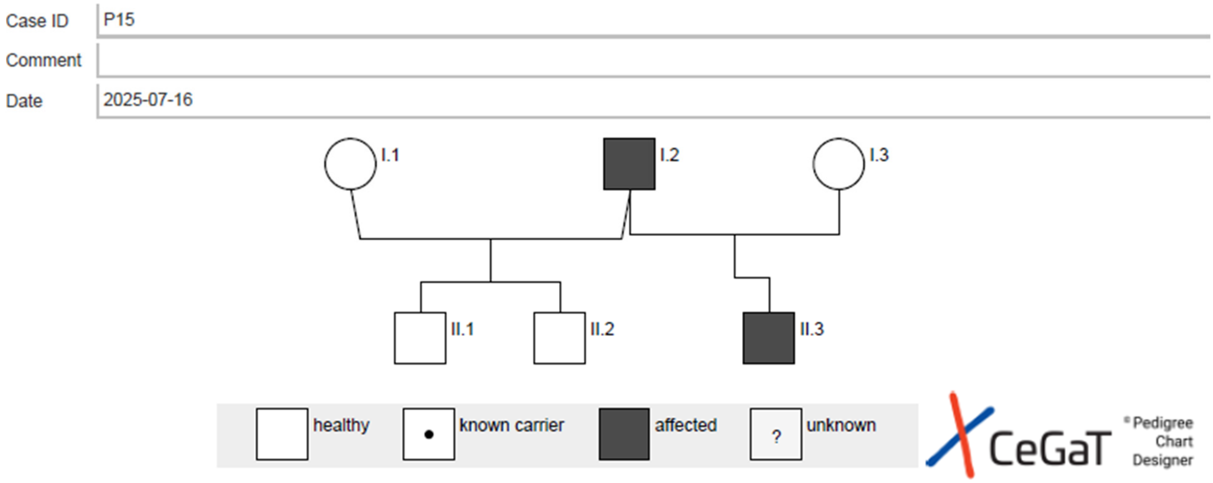

Case ID
P15
Comment
Date
2025-07-16
I.1
I.2
I.3
II.1
II.2
II.3
healthy
known carrier
affected
?
unknown
CeGaT
® Pedigree
Chart
Designer

D

Case ID	P16
Comment	
Date	2025-07-16

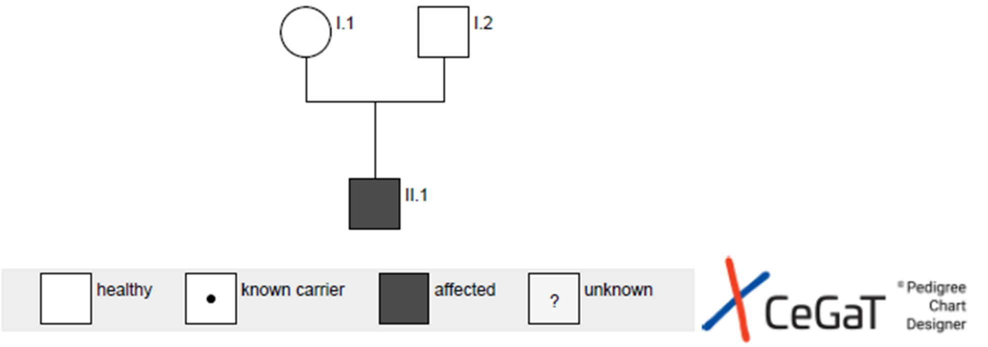

E

Case ID	P17
Comment	
Date	2025-07-16

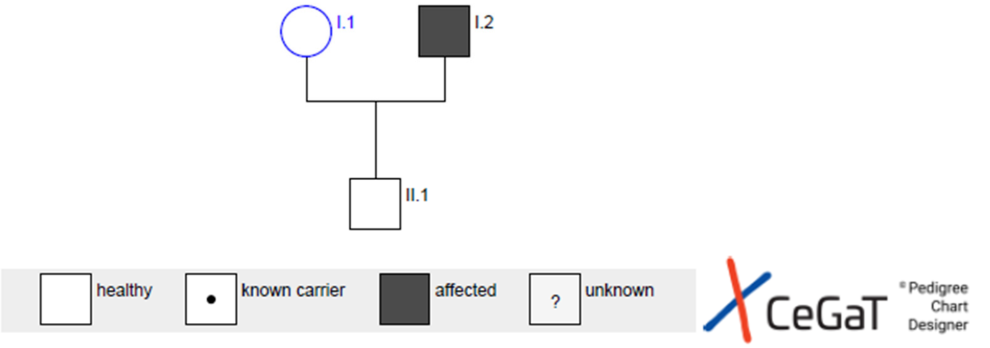

F

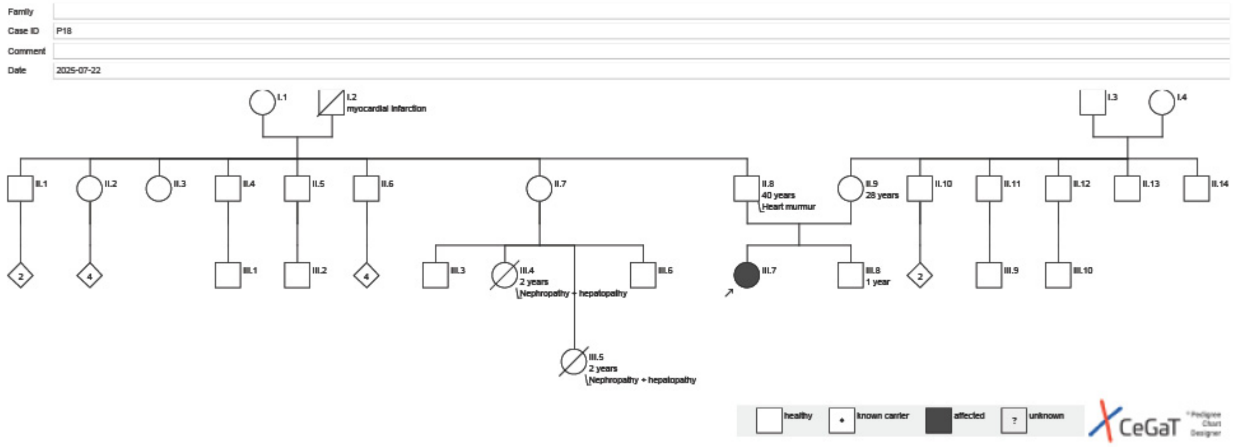

Figure S1. Selection of pedigrees of the cohort. A. Patient P2. B. Patient P3. C. Patient P15. D. Patient P16. E. Patient P17. F. Patient P18. Created with CeGaT Pedigree Chart Designer.
